# Collapsing Glomerulopathy: A Review by the Collapsing Brazilian Consortium

**DOI:** 10.3389/fmed.2022.846173

**Published:** 2022-03-03

**Authors:** Érico Murilo Monteiro Cutrim, Precil Diego Miranda de Meneses Neves, Marcos Adriano Garcia Campos, Davi Campos Wanderley, Antonio Augusto Lima Teixeira-Júnior, Monique Pereira Rêgo Muniz, Francisco Rasiah Ladchumananandasivam, Orlando Vieira Gomes, Rafael Fernandes Vanderlei Vasco, Dyego José de Araújo Brito, Joyce Santos Lages, Natalino Salgado-Filho, Felipe Leite Guedes, José Bruno de Almeida, Marcelo Magalhães, Stanley de Almeida Araújo, Gyl Eanes Barros Silva

**Affiliations:** ^1^University Hospital, Federal University of Maranhão, São Luís, Brazil; ^2^Clinical Hospital, University of São Paulo, São Paulo, Brazil; ^3^Clinical Hospital, Botucatu Faculty of Medicine of São Paulo State University, Botucatu, Brazil; ^4^Nephropathology Institute, Federal University of Minas Gerais, Belo Horizonte, Brazil; ^5^Department of Genetics and Postgraduate Program in Genetics, Ribeirão Preto Medical School, University of São Paulo, Ribeirão Preto, Brazil; ^6^University Hospital, Federal University of Paraíba, João Pessoa, Brazil; ^7^University Hospital, Federal University of Vale do São Francisco, Petrolina, Brazil; ^8^University Hospital, Federal University of Alagoas, Maceió, Brazil; ^9^University Hospital, Federal University of Rio Grande do Norte, Natal, Brazil; ^10^Laboratory of Genomic and Histocompatibility Studies, University Hospital, Federal University of Maranhão, São Luís, Brazil

**Keywords:** segmental and focal glomerulosclerosis, nephrotic syndrome (NS), renal biopsy, podocytes, glomerulopathy

## Abstract

Collapsing glomerulopathy (CG) is a clinicopathologic entity characterized by segmentar or global collapse of the glomerulus and hypertrophy and hyperplasia of podocytes. The Columbia classification of 2004 classified CG as a histological subtype of focal segmental glomerulosclerosis (FSGS). A growing number of studies have demonstrated a high prevalence of CG in many countries, especially among populations with a higher proportion of people with African descent. The present study is a narrative review of articles extracted from PubMed, Medline, and Scielo databases from September 1, 2020 to December 31, 2021. We have focused on populational studies (specially cross-sectional and cohort articles). CG is defined as a podocytopathy with a distinct pathogenesis characterized by strong podocyte proliferative activity. The most significant risk factors for CG include *APOL1* gene mutations and infections with human immunodeficiency virus and severe acute respiratory syndrome coronavirus 2. CG typically presents with more severe symptoms and greater renal damage. The prognosis is notably worse than that of other FSGS subtypes.

## Introduction

In many countries, focal segmental glomerulosclerosis (FSGS) comprises the main histological diagnosis in patients with nephrotic syndrome (NS) (1). However, FSGS is nowadays understood as a heterogeneously entity. Cases with more marked glomerular collapse patterns have been described since 1974 (2). In 1986, a notable article described six African-American patients with NS and severe renal damage. Histological evaluation demonstrated glomerular collapse and significant tubulointerstitial damage (3), and the follow-up was marked by rapid progression requiring renal replacement therapy (RRT). Weiss followed this series of patients—one of whom was later diagnosed with human immunodeficiency virus (HIV). In 1988, the renal biopsy findings of nine patients supported the association of HIV with glomerular collapse (4). Since then, collapsing glomerulopathy (CG) has been recognized as one of the main histological forms of HIV-associated nephropathy (5).

Further studies have reported a strong association between CG and *APOL1* gene mutations, especially among the black population of sub-Saharan Africa (6). In 2020, severe acute respiratory syndrome coronavirus 2 (SARS-CoV-2) infection showed a strong association with this histological pattern (7), which highlighted the need to improve our understanding about this disease.

## Epidemiology

The prevalence of CG varies widely between countries and may be difficult to assess because CG and FSGS are commonly analyzed together (8). Population-based studies in the United States, India, Pakistan, Macedonia, and Portugal proposed prevalence rates of 1.7, 0.75, 0.38, 1.7, and 0.29%, respectively (Table 1) (9–13). To the best of our knowledge, no population-based prevalence studies have been conducted in Brazil; however, the São Paulo Registry of Glomerulopathies indicates that 36% of FSGS cases comprise patients with CG (14).

**TABLE 1:** CG prevalence in populational studies.

Study	Country	Population	CG/FSGS	CG/PG	HIV+
De Abreu Testagrossa (14)	Brazil	48	48/131	48/525	0
Detwiller (15, 16)	United States	16	–	16/849	0
Haas (15)	United States	21	21/450	21/7420	0
Valeri (17)	United States	43	43/394	43/4073	0
Grcevska (12)	Makedonia	16	–	16/893	0
Laurinavicius (18)*	United States	60	–	–	18
Ferreira (13)	Portugal	18	18/413	18/6130	10
Mubarak (11)	Pakistan	10	–	10/2160	0
Ahuja (19)	India	30	–	30/3314	0
Laurin (20)	United States	61	–	–	0
Kanodia (10)	India	25	–	25/3335	0
Husain (21)	Saudi Arabia	31	31/173	–	0
Kukkul (22)**	United States	41	–	–	–

GC, collapsing glomerulopathy; FSGS, focal segmentar glomerulosclerosis; PG, primary glomerulopathies. *HIV+ and HIV- patients were compared to each other, without global results. **There were avalieted only patients from six months of 2015. **In this paper, there were included solely patients with 65 years old or older.

A survey conducted between 1979 and 1993 in the renal pathology center of Chicago suggested a significant increase in the proportion of CG among primary FSGS cases. In particular, the proportion of CG increased by 10, 20, and 24% in 1979–1985, 1985–1989, 1989–1993, respectively (15), which may be attributed to improvements in histopathologic diagnoses or changes in the epidemiological behavior of conditions such as HIV.

## Pathogenesis

### Cell Behavior

**FIGURE 1:**
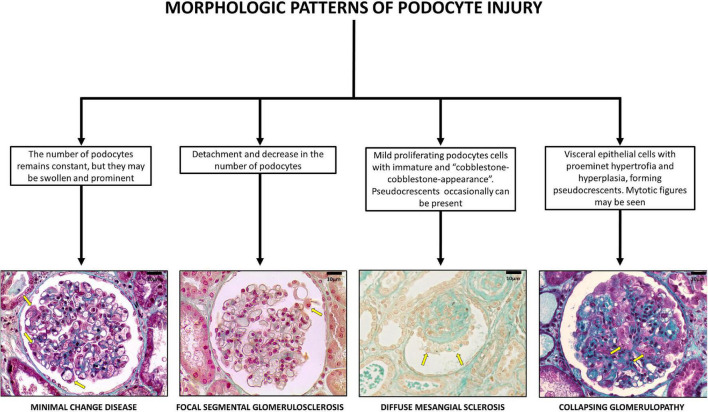
Glomerular pattern and podocytes morphological changes in human podocytopathies (bar = 10 μm).

From a histopathologic perspective, CG falls within the spectrum of podocytopathies (Figure 1). Podocytes are terminal cells that constitute an essential part of the glomerular structure. They consist of a cell body, primary, secondary, and tertiary podocyte processes, and interdigitations between these processes (8). The spaces between pedicels are filled with slit diaphragm proteins, which function as mechanical and electrical barriers for a plasma ultrafiltrate (23–25). The proteins that constitute the diaphragm are strongly anchored to the cytoskeleton of the podocytes (23) and comprise a strong functional part of the glomerular filtration barrier.

Barisoni (26) classified podocytopathies based on their morphological characteristics and pathogenesis. Minimal change disease presents with slit diaphragm involvement instead of podocyte damage, whereas FSGS presents with marked podocytopenia (27). In contrast, diffuse mesangial sclerosis (DMS) and CG are characterized by high proliferation rates (9). The proliferation rate is higher in CG than in DMS (26).

Although podocytes are usually considered as terminal differentiation cells, they have a marked loss of differentiation and high mitotic rates in CG. Shkeri utilized the western blot technique to analyze the gene expressions of several glomerulopathies and demonstrated that CG was associated with increased cell proliferation markers, such as T-telomerase, Ki-67 protein, and beta-cadherin 1; however, these findings were not observed in the other types of FSGS (28).

The human Wilms tumor 1 (WT1) gene is a tumor suppressor gene that spans ∼50 kb and consists of 10 exons. It encodes a protein that shares a high degree of structural homology with the early growth response family of transcription factors. Several lines of evidence suggest that WT1 is important for normal podocyte function (29). Overexpression of the Wilms tumor 1 (WT1) protein results in an increased telomerase function, which promotes podocyte cell differentiation and renal histological organization. Inhibition of the WT1 protein is associated with terminal phenotype loss and podocyte hyperplasia (28). In CG, WT1 inhibition results in podocytes that have similar gene expressions patterns with those of the glomerular parietal epithelium, which may represent a phenotypic return to the original cells that yielded the Bowman’s capsule epithelium (28). WT1 has been demonstrated to activate transcription of the podocalyxin gene. The integral membrane protein podocalyxin connects to the cytoskeleton of the podocytes and is implicated in maintaining the complex three-dimensional shape of the cells (29). Gene inhibition of podocalyxin and other proteins that contribute to the structure of podocytes and slit diaphragms by determining protein binding and local electrostatic forces as synaptopodin, glomerular epithelial protein 1, common acute lymphoblastic leukemia antigen, and the C3b receptor has also been documented in CG (24).

Other cells may also be associated with the pathophysiology of CG. Under hypoxic conditions, glomerular endothelial cells have been reported to secrete paracrine factors that modify podocyte structure (30). HIV-affected T lymphocytes are also involved in cell proliferation (31).

### Genetics

*APOL1*, which is located on the long arm of chromosome 22 (22q12 region), contributes to the pathogenesis of CG. It is responsible for forming high-density lipoproteins in different cell membranes and is associated with innate immunity. *APOL1* confers resistance to *Trypanosoma brucei rhodesiense*, the etiologic agent for African sleeping sickness. *APOL1* improves gene function and fights against infections through G1 (missense) and G2 (deletion of two amino acids) mutations. *APOL1* is also associated with several cell damage mechanisms, such as mitochondrial damage, lysosomal degranulation, and cell pore formation (6). Lysosomal degranulation plays a particular role in destroying the cell membranes of foreign pathogens (32).

*APOL1* mutations are correlated with the distribution of sleeping sickness. As such, these mutations are quite prevalent in sub-Saharan Africa, reaching over 40% in countries such as Ghana and Nigeria (6). In several countries including Brazil, *APOL1* mutations are considered as risk factors and determinants of chronic kidney disease (CKD). A national case-control study demonstrated that these mutations are associated with an odds ratio of 10.95 for progression to CKD compared to controls. *APOL1* mutations have also been associated with an increased indication for hemodialysis within a mean period of 12 years or earlier (33).

Other genes have been implicated in the pathogenesis of CG. The *COQ2* gene (4q21.23) encodes the mitochondrial protein CoQ1, which plays a role in some neurologic, muscle, and renal syndromes. Mitochondrial gene mutations contribute to the proliferation of a poorly differentiated podocyte profile, which results in CG (34, 35). In contrast, the *MHY9* gene is responsible for synthesizing myosin microfilaments that maintain the podocyte structure and filtration barrier. Among patients with HIV, *MHY9* gene mutations increase the risk of progression to CG by 4–8-fold (36).

### Etiologies

**FIGURE 2:**
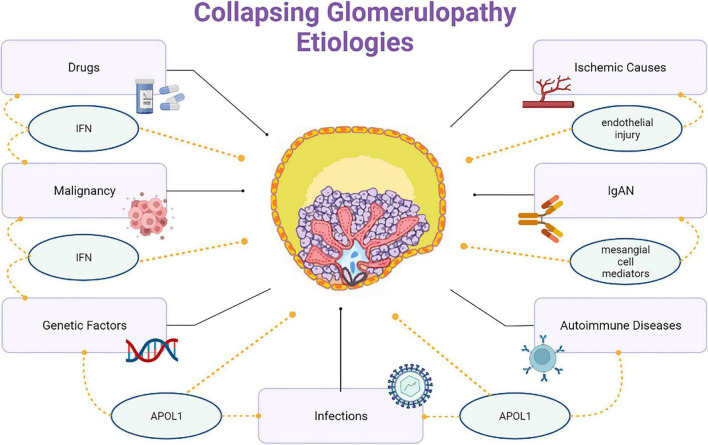
Collapsing glomerulopathy etiologies.

Barisoni proposed a classification scheme for podocytopathies and characterized CG as idiopathic/primary, genetic, and reactive/secondary (26) (Figure 2). The primary conditions that cause CG are highlighted here and classified according to the nature of injury, as follows:

(1) Infectious etiologies like HIV, T-cell lymphotropic virus, hepatitis B virus (HBV), hepatitis C virus (HCV), Epstein-Barr virus, cytomegalovirus (CMV), parvovirus B19, dengue, Zika virus, Chikungunya, tuberculosis, visceral leishmaniasis, filariasis, and SARS-COV-2.
(2) Autoimmune etiologies including systemic lupus erythematosus (SLE), adult Still’s disease, temporal arteritis, mixed connective tissue disease, and Behçet’s disease.
(3) Neoplastic etiologies, such as multiple myeloma, acute monoblastic leukemia, natural killer cell leukemia, and acquired hemophagocytic syndrome.
(4) Drug-related etiologies like bisphosphonates, interferons, anabolic steroids, heroin, valproic acid, and anthracyclines.
(5) Ischemic etiologies, that include thrombotic microangiopathy (TMA), atheroembolic disease, sickle cell anemia, and thromboembolism by hydrophilic polymers.

Collapsing glomerulopathy can also coexist with other nephropathies, such as immunoglobulin A (IgA) nephropathy, membranous glomerulopathy, and other histological types of FSGS and DMS.

Several case reports are published annually that link CG to infectious diseases. Chandra (37) proposed the following criteria to assess the causal link between infection and CG: (1) CG demonstration in multiple cases of viral disease; (2) clear demonstration of CG lesions, including glomerular tuft collapse and changed podocyte phenotype; (3) demonstration of viral proteins or nucleic acids in glomerular cells, especially in podocytes; and (4) experimental demonstration (in animal models) of some (or all) CG findings.

Collapsing glomerulopathy is the most common morphological pattern in HIV nephropathy (38). The increased incidence of HIV infection has been identified as a cause for the proportionate increase in the cases of CG (17). Disease activity characterized by a high viral load and low CD4 lymphocyte count results in greater renal damage (5); however, the virus has also been detected in the renal tissue of patients with undetectable viral loads (31). Non-structural HIV proteins, such as viral protein R and negative factor, promote cell cycle dysregulation, which stimulates podocyte proliferation. Other factors (such as chronic inflammation) are associated with the genetic predisposition for *APOL1* and *MHY9* mutations, which result in CG (31). In some studies, the prevalence of HIV infection in patients with CG ranged from 30 to 55% (13, 18).

Cytomegalovirus infection is associated with immunosuppression, but it can also occur in immunocompetent patients and progress to CG, even in the acute phase of the disease. Specific treatment is associated with several benefits for prognosis in this population (37, 39–41).

Arboviruses were recently identified as an important causative factor for CG, particularly in Brazil. Eight of 13 CG biopsy samples obtained from the first half of 2016 from a large Brazilian kidney biopsy center were positive for arbovirus, wherein six samples were positive for dengue, one for Zika, and one for a concomitant infection; only one case had *APOL1* mutations. These findings suggested that direct viral action in tissues may be associated with other risk factors, such as G1 and G2 mutations (42, 43).

The association between SARS-CoV-2 infection and CG was initially demonstrated in a series of autopsies (7). A recent systematic review of 59 studies reporting COVID-19 related histopathological diagnoses from kidney biopsy identified CG as the most common finding, followed by acute tubular injury and trombotic microangiopathy (44). Various mechanisms of AKI secondary to COVID-19 have been proposed—from direct intrarenal infection to dysregulation of the renin-angiotensin-aldosterone system, to altered hemodynamic control, coagulation and cytokine homeostasis (44). Although the association is multifactorial, it has been emphasized the influence of the hyperactive inflammatory process and participation of circulating interferons (44) and direct infection appears highly unlikely to play a significant pathogenic role.

*APOL1* mutations have been detected in many patients with COVID-19, which suggested that the virus is a potential secondary trigger for glomerular damage (45). In fact, studies have confirmed the strong association of CG and non-collapsing podocytopathies with concurrent or recent COVID-19 in patients with APOL1 high-risk alleles (44–46).

Systemic lupus erythematosus can trigger CG as an extreme form of lupus podocytopathy in the absence of other lupus nephritis patterns (47). CG may also result from the association of SLE with other risk factors, such as black ethnicity and *APOL1* mutations. CG is occasionally present during the diagnosis of SLE (47), with low levels of therapeutic response.

Collapsing glomerulopathy can be drug-induced; bisphosphonates, especially pamidronate and zoledronic acid, inhibit the mevalonate synthesis pathways, which are essential for cell differentiation. This triggers podocyte proliferation and progression to CG (48). Synthetic interferons (which are used to treat some infectious, autoimmune, and neoplastic diseases) can result in *APOL1* overexpression in the glomerular epithelium, which triggers podocyte damage. This effect is evidenced by the presence of tubuloreticular inclusions on electron microscopy (48). Illicit drugs, such as cocaine and heroin, are also associated with CG. The most probable mechanism involves ischemic glomerular damage from oxidative endothelial damage, accelerated atheromatosis, and direct vasoconstriction (49).

Acquired Hemophagocytic Syndrome results from a hyperactive immune system that develops secondary to infectious diseases or lymphatic hematological neoplasms. Excessive T lymphocyte and circulating cytokine activation can promote podocyte proliferation and progression to CG (50). Monoclonal gammopathies can also encourage progression toward CG. Histological analysis demonstrates diffuse Ig chain deposits, with glomerular collapse resulting from the deposition of extracellular elements instead of from podocyte proliferation (51). Once triggered, CG progresses independently of the underlying disease. Further, while the underlying gammopathy may go into remission, CG can continue to worsen until RRT becomes necessary (52).

Collapsing glomerulopathy is also associated with diseases characterized by microvascular damage, such as sickle cell anemia, intravascular hemolysis syndromes, drug reactions (calcineurin inhibitors), malignant arterial hypertension, and antiphospholipid syndrome. A total of 53 histological samples that demonstrated TMA were evaluated. These samples were acquired from 33 patients with FSGS, 19 of whom had CG (30). Glomerular ischemia resulted in the loss of podocyte differentiation and reduced cell proliferation. The population in this study was predominantly of white ethnicity and was characterized by a lower incidence of nephrotic proteinuria.

Other conditions, such as hypertensive disorders of pregnancy (HDP), have also been implicated as a trigger for CG. HDP are characterized by diffuse endothelial damage and possible glomerular ischemia. Kidney biopsy often shows association between CG and TMA (46).

## Histology

**FIGURE 3:**
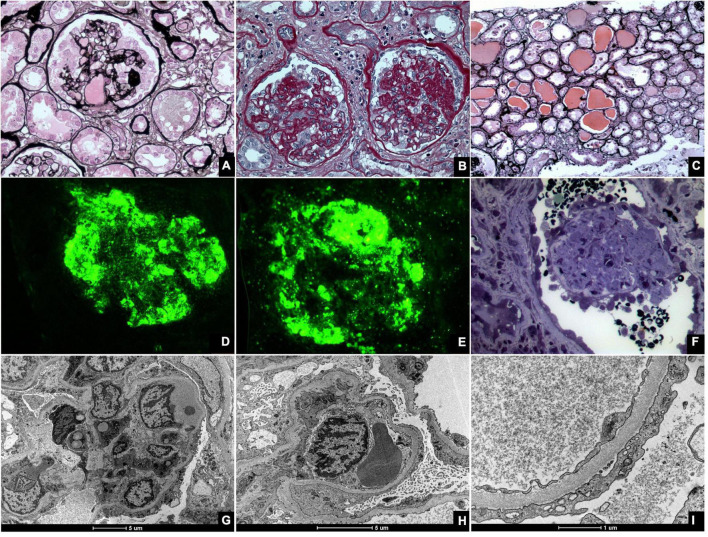
Kidney biopsies of collapsing glomerulopathy. **(A,B)** Periodic Acid Schiff (PAS) and Jones Methenamine Silver (JMS) (40×), respectively show intense podocyte hyperplasia and glomerular tuft collapse. **(C)** JMS (20×) exhibits microcytic transformation of distal convoluted tubules with accumulations of hyaline material inside of those. **(D,E)** Fluorescence microscopy (40×) shows, respectively, IgM and C3 trapping in areas of collapse/sclerosis. **(F)** Semi-fine stained in Toluidine Blue (63×) with collapse of the entire glomerular tuft and hyperplasia of podocytes and dilated Bowman’s space. **(G,H)** Transmission electron microscopy contrasted with Osmium Tetroxide, Lead Citrate and Uranyl in block shows capillary loop collapse with hyalinosis in addition to diffuse fusion and flattening of the pedicels associated with microvillous transformation. **(I)** Electron microscopy tubes contrasted with osmium tetroxide, lead citrate, and uranyl in block with detail of disorganization of the cytoskeleton in the podocyte cytoplasm, with extensive effacement of the pedicels.

Although initially treated as a single entity, FSGS encompasses several distinct histological patterns. FSGS is heterogenous in terms of etiology, histological characteristics, clinical presentation, treatment response, and prognosis. For this reason, a classification of FSGS histological subtypes was proposed in 2004 (53). The diagnosis of CG requires the presence of at least one glomerulus with segmental or global collapse and podocyte hypertrophy and hyperplasia (Figure 3). CG can occur with other morphological subtypes, and a single characteristic lesion is adequate for a diagnosis of CG.

Light microscope shoes partial or total obliteration of the lumen of the glomerular capillaries secondary to podocyte hypertrophy and hyperplasia (53, 54). Podocyte crowns may contain intracytoplasmic deposits, which denote protein reabsorption. Pseudocrescents, which resemble glomerular crescents, may temporally precede glomerular sclerosis (27). Other findings, such as glomerulomegaly, hyalinosis, hypercellularity, and adhesions, are unusual but more common in the final stages of the disease (55).

As a rule, tubulointerstitial damage in CG is more intense than in other FSGS subtypes (17). Important specific findings include tubular dilatation with molding and the formation of tubular microcysts (10, 17). Tissue and lymphatic macrophages, specifically CD4 and CD8, can also be found in interstitial infiltrates (10).

Immunofluorescence (IF) findings are non-specific and, in many cases, negative. If present, they usually comprise granular or mesangial IgM and C3 deposits. IF may suggest associated conditions such as Berger’s disease, which is characterized by mesangial IgA deposits (54), or SLE, which is associated with several antibody deposits.

Electron microscopy (EM) is mostly unnecessary; however, it can demonstrate collapsed and ruptured capillary membranes and swollen, hypertrophic, and/or hyperplastic podocytes (54). EM may also identify intracellular inclusions that are often associated with HIV or SLE. In these conditions, the podocyte processes are greatly compromised with loss of glomerular filtration barrier integrity.

## Clinical Presentation

The most frequent clinical presentation of CG is massive, severe proteinuria associated with pure NS. Patients may present with hypertension, lipiduria, and hematuria on urinalysis. A significantly high number of patients present with renal failure on admission and rapidly progress to end-stage renal disease (ESRD) (13, 17, 20, 27, 56).

Collapsing glomerulopathy demonstrates no sex predilection, but preferentially affects patients of African descent. The disease also often affects young adult patients and has a varying predominance in the pediatric age group (10, 13, 17, 19, 20, 57).

The normal kidney dimensions are usually preserved in CG and presents as in other conditions, such as diabetic nephropathy, polycystic kidney disease, and amyloidosis. Ultrasonography may demonstrate normal or increased renal size and hyperechogenicity, which may be due to edema, fibrosis, and tubulointerstitial infiltrates resulting from the rapid progression to ESRD and absence of renal parenchymal contraction (17).

## Treatment

The treatment of CG consists of the following: (1) targeted therapy for disorders resulting from NS, such as dyslipidemia, hypertension, and edema; (2) treatment of the underlying disease when CG is associated with other conditions; and (3) immunosuppressive therapy.

According to the Kidney Disease Improving Global Outcomes guidelines on the management of glomerulopathies (58, 59), the measures to control complications associated with NS consist of edema control through a low-sodium diet (<2 g/day), fluid restriction, diuretic therapy, and, if necessary, hemodialysis or ultrafiltration. Systolic blood pressure should be maintained at <120 mmHg with angiotensin-converting enzyme inhibitors or angiotensin II receptor blockers, unless renal function worsens on these medications (60).

The severity of dyslipidemia (DLP) in patients with nephrotic syndrome (NP) is proportional to the degree of proteinuria. Once the proteinuria in patients with GC can be massive, the serum levels of cholesterol and its fractions as well as lipoproteins can reach high elevated values. The potential contributors to DLP genesis in patients with nephrotic syndrome are the patient’s diet, use of drugs (such as corticosteroids, calcineurin inhibitors, and mTOR inhibitors), in addition to genetic predisposition (61, 62). According to the new KDIGO 2021 Clinical Practice Guideline for the Management of Glomerular Diseases (60) in patients with NP the DLP should be controlled, especially in those ones with other cardiovascular risk factors (e.g., hypertension and diabetes) and/or diseases that respond poorly to stabilized therapy.

As non-pharmacological measures, lifestyle changes (diet, smoking cessation, weight loss, and physical activity) must be encouraged in all patients. The tratment with statins as a first-line treatment should be indicated in patients at increased risk of atherosclerotic cardiovascular disease, such as those with GFR <60 ml/min/1.73m^2^ and/or albuminuria >30 mg/g, in addition to treating infectious/inflammatory diseases that contribute to increased cardiovascular risk. For patients who do not tolerate the use of statins or cannot achieve the recommended lipid levels targets, other drugs as bile acid chelators, fibrates, nicotinic acid, ezetimibe, PCSK9 inhibitors and lipid apheresis can be used.

Special attention should also be given to the risk of thrombotic events; prophylactic anticoagulation should be considered for susceptible populations. Immunocompromised patients should be given full immunizations; tested for HIV, HBV, HCV, tuberculosis, and syphilis; and initiated on strongyloidiasis and pneumocystis prophylaxis.

When an underlying etiology is identified, such as drug-related, infectious, genetic, autoimmune, and other causes, the patient should undergo specific therapy, when available. In drug-induced cases, the offending drug must be discontinued whenever possible (63). In cases associated with infections, specific antimicrobial treatments can reduce CG progression or even promote remission. Some HIV-associated cases demonstrate 38% delay in the progression to ESRD following the initiation of antiretroviral therapy (ART) (64); however, other studies proposed that up to 50% of patients progress toward the initiation of RRT despite adequate treatment (65). Nevertheless, HIV-associated nephropathy should always be treated with ART because it is very effective at controlling the disease (66).

Intravenous Ig and cidofovir can be used to treat parvovirus B19 infection, which is particularly important from the perspective of renal transplantation (67). CMV infections should be treated with ganciclovir, which also promotes CG remission and renal function improvement (39).

Immunosuppressant therapy was administered to most patients. While there are no specific recommendations for CG, the immunotherapy guidelines for CG are extrapolated from the FSGS protocol (20). Several types of immunosuppressants have been proposed (16–18, 20), but the initial therapeutic regimen consists of high-dose of oral corticosteroids for 4–16 weeks or until remission is achieved. Calcineurin inhibitors or cyclophosphamide are considered second-line medications for patients that exhibit corticosteroid resistance, corticosteroid dependence, or frequent relapses; they should be given for at least 12 weeks. Other immunosuppressants (such as rituximab and mycophenolate mofetil) are indicated for patients who do not respond to other immunosuppressive regimens. Patients with extensive kidney damage may choose to forego treatment for CG because of the lack of benefits and risks associated with immunosuppression therapy.

## Prognosis

Collapsing glomerulopathy has a poor prognosis. Most cases present with refractory proteinuria, severe loss of renal function, and progression to permanent RRT (15, 53, 54). While other FSGS subtypes have a renal survival time of 62.5 months, CG has a renal survival time of 13 months (17). Table 2 summarizes the treatment data and prognoses collected by the studies that examined patients with CG.

**TABLE 2:** CG patients’ treatment and follow-up data.

Authors	Total	Treated patients	Treatment	Renal Outcome	Need for RRT (%)
Valeri et al. (17)	43	35	26 CS	No remission	51
			6 CP	1 TR	
			3 CI	1 PR, 1 PR	
Laurinavicius et al. (18)	42	37	23 CS	2 TR, 7 PR	73.33
			3 CP	No remission	
Raja et al. (68)	22	22	1 AR	No remission	36.4
			16 CS	No remission	
			4 CS + TAC	3TR, 1 PR	
			1 CS + CP	No remission	
Grcevska et al. (12)	16	15	7 CS	No remission	100
			8 CS + CP	No remission	
Detwiler et al. (16)	16	5	4 CS	1 TR	35.71
			1 CS + CP	No remission	
Chun et al. (69)	40	25	40 CS	6 TR, 10 PR	43
Singh et al. (70)	6	6	6 CS	No remission	66.7
			2 CP after CS	2 TR	

*ARC, angiotensina receptor II blocker; CS, corticosteroids; CI, calcineurin inhibitors; CP, cyclophosphamide; RRT, renal replacement therapy; TR, total remission; PR, partial remission; TAC, tacrolimus.*

## Conclusion

The current literature demonstrates that CG is a distinct clinicopathological entity that results from complex interactions between intrinsic (genetic mutations) and extrinsic (especially infections) factors. CG presents at more advanced stages and with poorer renal function, which emphasizes that it must be evaluated properly, especially in developing countries where risk factors, such as *APOL1* mutations and viral infections, are more common.

The prognosis of CG is notably worse than that of the other FSGS subtypes. A large number of young patients with CG progress to permanent RRT. Larger longitudinal studies are needed to assess the factors associated with CG in different populations and determine the clinical and histological elements that contribute to the final prognosis.

This article was written by the Collapsing Brazilian Consortium, an initiative in Brazil that brings nephrologists and pathologists together to study CG.

## Author Contributions

ÉC, PN, MC, and GS conceived and designed the analysis, performed the analysis, and wrote the manuscript. DW, MMu, FL, OG, RV, DB, JL, NS-F, JA, MMa, and SA contributed data or analysis tools and performed the analysis. AT-J contributed data or analysis tools, performed the analysis, and wrote the manuscript. FG contributed data or analysis tools, performed the analysis. All authors contributed to the article and approved the submitted version.

## Conflict of Interest

The authors declare that the research was conducted in the absence of any commercial or financial relationships that could be construed as a potential conflict of interest.

## Publisher’s Note

All claims expressed in this article are solely those of the authors and do not necessarily represent those of their affiliated organizations, or those of the publisher, the editors and the reviewers. Any product that may be evaluated in this article, or claim that may be made by its manufacturer, is not guaranteed or endorsed by the publisher.
